# Spasticity Treatment During COVID-19 Pandemic: Clinical Recommendations

**DOI:** 10.3389/fneur.2020.00719

**Published:** 2020-06-23

**Authors:** Alessio Baricich, Andrea Santamato, Alessandro Picelli, Giovanni Morone, Nicola Smania, Stefano Paolucci, Pietro Fiore

**Affiliations:** ^1^Physical and Rehabilitation Medicine, Department of Health Sciences, Università del Piemonte Orientale, Novara, Italy; ^2^Physical and Rehabilitation Medicine Department, Università di Foggia, Foggia, Italy; ^3^Neuromotor and Cognitive Rehabilitation Study and Research Centre, Department of Neuroscience, Biomedicine, and Movement Sciences, University of Verona, Verona, Italy; ^4^Santa Lucia Foundation (IRCCS), Rome, Lazio, Italy

**Keywords:** pandemic (COVID-19), COVID-19, intrathecal baclofen, Botulinum Toxin, rehabilitation, spasticity, Coronavirus

## Introduction

Spasticity is a symptom that describes involuntary muscle hyperactivity in the presence of central paresis due to several neurological conditions (1). It can consist of various clinical forms, and it has been reported (2, 3) that spasticity showed a prevalence of 28–38% in patients with stroke, 41–66% in patients with multiple sclerosis, 13% in patients with traumatic brain injury, and up to 80% of children with cerebral palsy.

Spasticity can affect quality of life, impair function and heighten economic burden (4, 5), and it could be associated with several complications, including contractures, pain, fall risk, pressure ulcers, and infections (6). In addition, caregivers of patients affected by spasticity are more likely to experience anxiety and depression (7).

Spasticity management aims to reduce its negative impact on patients and carers and to prevent irreversible soft-tissue changes and tendon contractures by maintaining muscle length and normalizing limb positioning (8, 9).

Identifying and treating clinically relevant spasticity is key to decreasing patients' impairments (10, 11). Interventions must be tailored to meet the problems faced by the person and their goals, including focal (e.g., chemodenervation with Botulinum Toxin, chemical neurolysis) and general treatments (e.g., oral antispasticity drugs, cannabinoids, intrathecal baclofen) (10, 11). Besides, a multidisciplinary team including doctors, physiotherapists, occupational therapists and nurses, is required: in fact, other physical modalities can optimize the effect of pharmacologic treatment (e.g., stretching, splinting, postural management, exercise, electrical stimulation, casting, splinting, extracorporeal shock waves, body vibration) (10, 12).

Noteworthy, it should be pointed out that patients affected by spasticity require periodic access to the health care facilities.

In particular, Intrathecal baclofen infusion (ITB) systems, proposed in case of severe generalized spasticity, imply a close follow-up for safety purposes; notably, ITB pump refill is a programmed procedure that requires regularity in its execution, and that cannot be postponed due to the risk of withdrawal symptoms (13).

Again, Botulinum toxin Type A (BoNT-A), the gold standard for focal spasticity treatment, requires a regular administration (every 3–6 months) in order to maintain the clinical effect (14); moreover, BoNT-A must be proposed by a multidisciplinary team, since optimal treatment involves physical therapy in conjunction with intermittent pharmacological treatment (14, 15).

It is well-known that when spasticity worsens, patients may experience a variety of symptoms (10). In particular, prolonged suspension can potentially accelerate the morphological alterations connected with spasticity (e.g., myotendinous and joint contractures, pain) which could potentially cause a long-term negative impact on the patients' level of activity and participation, as well as to a deterioration in their quality of life (8, 9).

The recent reorganization of non-urgent clinical activities, connected to the emergency generated by the COVID-19 pandemic, has also significantly involved the treatment of patients with spasticity.

As per institutional indications, most of these activities have been suspended or postponed (16, 17).

This situation, necessary in consideration of the pandemic, has nevertheless exposed patients suffering from spasticity to the risks connected to the interruption of the treatment as described above.

Based on these considerations, it seems reasonable to continue planning the spasticity treatment, carefully monitoring those that cannot be delayed.

However, several factors must be taken into account to guarantee both patients' necessary care and indications for minimizing a further spread of the pandemic.

For this purpose, an *ad-hoc* treatment protocol is summarized in the next section.

## Clinical Recommendations

Some of these aspects are part of the general indications for patients' access to healthcare facilities (18). Still, some specific elements must consider the patients' characteristics (19) and the specific settings where the treatments are carried out (16, 17).

### Inpatient Facilities

In this case, spasticity treatment is part of the rehabilitation program of the patient hospitalized for this purpose.

The hospital organization must consider the general indications for the containment of the infection (18, 20); therefore, all the appropriate procedures must be put in place to avoid exposing the patient to the risk of contracting COVID-19 (21), and in particular:

- adequate clinical monitoring of patients to identify clinical signs of potential COVID-19 onset (18)
- adequate use of personal protective equipment (PPE) about the procedures and clinical characteristics of the patients (18, 22, 23)
- monitoring of the health status of involved staff (18)
- training of staff and patients on compliance with hygiene rules (18)
- availability and easy retrieval of suitable indications (e.g., explanatory material distributed in hospital areas) and material (e.g., hand sanitizing gel) (18)
- blocking (or severe limitation) of access to visitors (18).

### Outpatient Facilities

In this case, since the patient's access to the hospital or outpatient facility takes place from the outside, it is necessary to consider a series of procedures to ensure the safety of the patient and operators (24).

**Figure 1:**
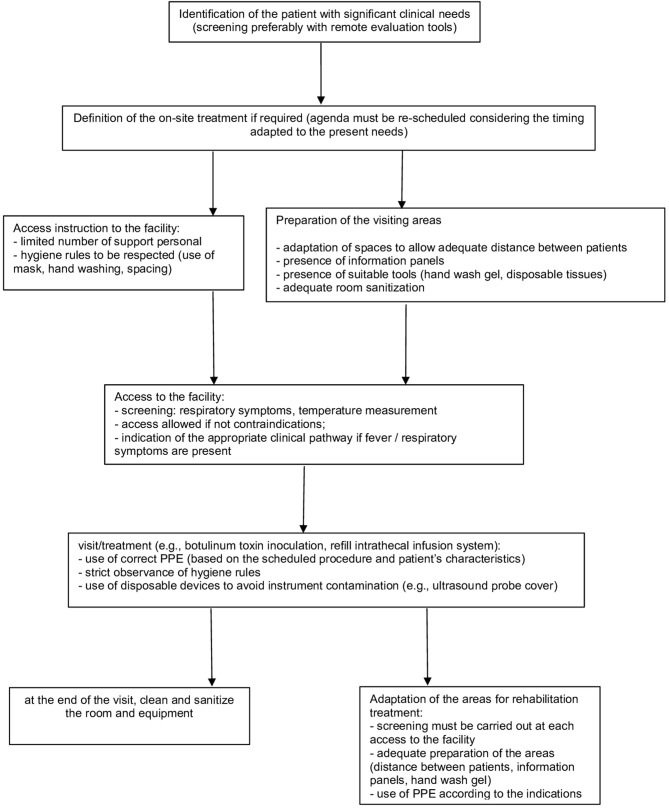
Outpatient management of spasticity treatment.

In particular, several aspects must be considered (Figure 1).

#### Patient Selection

- in consideration of current government indications (25), it remains a rule of good clinical practice to limit access to only patients for whom the treatment cannot be postponed (e.g., repetition of treatment with BoNT-A for significant reduction of autonomy consequent to the recovery of spasticity; ITB refill or follow up) (26)
- the use of telephone screening tools that allow remote pre-assessment is recommended to coordinate patient access to facilities: this is to facilitate the assessment of the patient's clinical needs and to monitor any presence of suggestive symptoms of COVID-19 or to identify any contact with other affected subjects; for this purpose, video call tools, where available, can also be supportive for an initial, albeit limited, clinical evaluation (17, 24).
- Alternatively, in this perspective, progressive implementation of suitable tools (video call programs, the supply of motion sensors) that can support the clinical evaluation remotely by the clinician is desirable, to monitor the situation of patients by reducing the number of accesses at health facilities; for this purpose, it is necessary to use the available technological resources to identify the most suitable tools. At the same time, it is also necessary to guarantee proper classification and financial rewards of these services (27).
- in case of previous BoNT-A treatment (14), we suggest considering a clinical assessment in the health facility if two or more of these issues are present at screening:
  Last inoculation date with BoNT-A > 3 months (yes/no)
  Increased spasticity in the muscles previously treated with BoNT-A, which can affect the patient's function or autonomy (yes/no)
  Presence of hypertonus in untreated muscles, which can affect the patient's function or autonomy (yes/no)
  Severe degree of spasticity conditioning a potential risk of long-term damage (e.g., myotendinous retractions) (yes/no)
  Significant presence of pain, potentially related to spasticity (yes/no)
  Impossibility of wearing orthoses/aids in use due to the presence of spastic hypertonicity (yes/no).
- We also suggest to carefully consider each of the above-reported points on patients treated with ITB due to the potentially life-threatening risk of pump emptying or malfunction (13).

#### Access to the Health Facility

Set a screening station at the entrance to identify subjects potentially affected by COVID-19 (e.g., targeted medical history, contactless temperature measurement) to minimize the risk of exposure to COVID-19; prepare an adequate clinical pathway in case of suspected infection (e.g., SARS-COV-2 swab test, according to the local guidelines) (18, 24)
verify that all staff are trained to recognize possible clinical signs compatible with COVID-19 and that they can provide the correct indications to the patient (18).

#### Health Service Provision

- The provision must be adapted to the general indications (24), with attention to specific aspects of the treatment of patients affected by spasticity.
- It may be necessary to remodel the interventions' planning to allow the implementation of all the appropriate procedures. In particular, patients must be properly scheduled in order to avoid gatherings of people (24)
- Reorganization of waiting rooms (18, 24, 28, 29):
  prepare the presence of information panels relating to COVID-19, highlighting the standards to be respected
  prepare the presence of tools such as hand wash gel and disposable tissues
  there must be as few people as possible in this area: where necessary, the service provision must be re-modulated. In particular, access to carers must be limited
  consider the adaptation of common areas to allow an adequate spacing of patients (>1 m) (28).

#### Adaptation of Treatment Procedures

**Figure 2:**
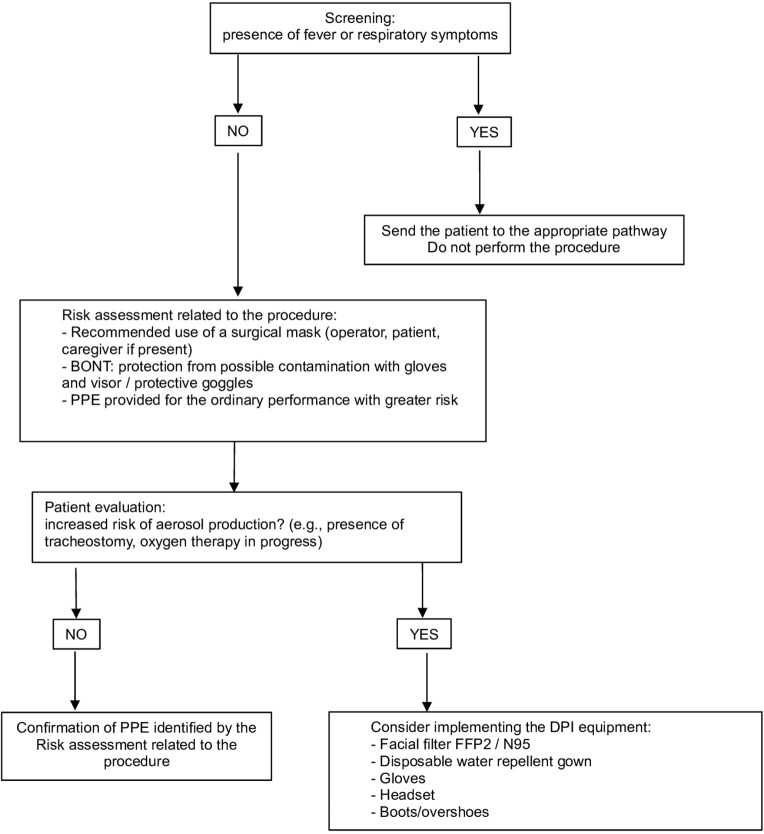
Personal Protection Equipment (PPE) management: risk assessment related to the procedure and patient characteristics.

- Considering the need to distance patients, the possible use of PPE with relative dressing/undressing procedures and the time required for the room cleaning, it is reasonable to set an agenda with scheduled appointments adapted to these needs (18, 22, 23)
- the use of correct PPE must be planned based on the scheduled procedure and the characteristics of each patient (Figure 2); in consideration of the potential risk of contagion even in asymptomatic subjects, the use of a surgical mask by both the healthcare professional and the patient is mandatory to limit the spread of the virus (18, 22, 23)
- strict observance of the usual hygiene rules (e.g., hand washing) is necessary to minimize the risk of virus transmission (18, 28–30)
- cleaning and sanitizing of instrumentation and environments are mandatory (18):
  - the environmental sanitation procedures must be implemented as per institutional or company indications by the dedicated staff, equipped with the appropriate PPE
  - in general, surfaces frequently touched by a large number of people (such as doorknobs, chairs, desks) must be cleaned at least daily and if possible more frequently; the use of regular detergents can be considered sufficient if there has been no contact with confirmed or suspected COVID-19 patients
  - However, it is conceivable to arrange additional cleaning of the surfaces and devices used during the procedures between one patient and another (e.g., ultrasound or electrical stimulation devices in injections with botulinum toxin, examination table). For this purpose, after removing any visible traces, a suitable product should be used wherever possible. Current evidence suggests (31) the use of a standard detergent associated, where possible, with a virucidal product or sodium hypochlorite 0.05% or ethanol 70%
  - the use of disposable devices that avoid contamination of the devices is desirable (e.g., probe cover when using ultrasound for injection procedures with BoNT-A).

#### Adaptation of Areas for Rehabilitation (if Applicable)

As previously stated, spasticity treatment requires multidisciplinary management. In particular, adjunctive treatment might improve the clinical effect of BoNT-A, and they should be applied in the health care facility immediately after the BoNT-A injection (12).

However, in order to minimize the risk during COVID-19 pandemic, several issues must be carefully considered:

- it must be highlighted that the screening procedures must be implemented at each access to the facility (24)
- we suggest that the rules of distancing between patients (>1 m) (28) must also be applied in the organization of the areas where the patient's rehabilitation treatment takes place (e.g., gyms, areas dedicated to the occupational therapy)
- the correct use of PPE for the staff involved must be considered based on the patient's characteristics (18, 22, 23)
- consider information panels and provide suitable material (e.g., hand wash gel) within the area (18, 24)
- prepare adequate plans for cleaning and sanitizing rooms and tools; in particular, attention must be paid to cleaning the equipment used by patients (e.g., electrical stimulation devices, dedicated equipment, and machinery, beds) (18, 31)
- if appropriate, exposure risk should be limited by implementing communication technologies which can support remote rehabilitation treatment (27, 32).

## Conclusions

The treatment of the patients suffering from spasticity, while not showing the characters of urgency except for some procedures such as ITB refill or monitoring, is worthy of particular attention in this phase of the COVID-19 pandemic.

It must be highlighted that this prolonged suspension of deferred activities has potentially exposed many patients to the disabling consequences of untreated spasticity.

Given these aspects, close monitoring of patients is recommended in order to plan an adequate schedule for the resumption of patients' treatment, in compliance with the rules for reducing the spread of the COVID-19 pandemic.

The use of remote assessment tools can support the identification of patients who require treatment in a short time to prevent the onset of complications that may further limit their level of activity and participation.

Looking ahead and considering the foreseeable need to adopt these precautions in the medium term, using these technologies can also allow adequate planning of patients' follow-up and rehabilitation treatment.

## Author Contributions

AB: conception of the document, document draft and revision. PF, SP, NS, GM, AP, and AS: conception of the document and document revision. All authors contributed to the article and approved the submitted version.

## Conflict of Interest

The authors declare that the research was conducted in the absence of any commercial or financial relationships that could be construed as a potential conflict of interest.
